# Verbal Reasoning Impairment in Parkinson's Disease

**DOI:** 10.1155/2022/3422578

**Published:** 2022-12-10

**Authors:** Antonina Luca, Giulia Donzuso, Concetta D'Agate, Claudio Terravecchia, Calogero Cicero Edoardo, Giovanni Mostile, Giorgia Sciacca, Alessandra Nicoletti, Mario Zappia

**Affiliations:** ^1^Department of Medical, Surgical Sciences and Advanced Technologies “G.F. Ingrassia”, University of Catania, Catania, Italy; ^2^Oasi Research Institute-IRCCS, Troina, Italy

## Abstract

**Background:**

The aim of this study was to assess verbal reasoning (VR) functioning in patients with Parkinson's disease (PD) and healthy controls (HCs).

**Methods:**

The non-demented PD patients and HCs matched by age and global cognition were enrolled in this study. VR was assessed with the verbal reasoning test (VRT), total score, and subsets.

**Results:**

Eighty-seven PD patients (51 men; mean age 63.8 ± 7.9 years) and 87 HCs (46 men; mean age 63.7 ± 8.0 years) were enrolled. At univariate analysis, PD patients presented a significantly lower score in the VRT subset classification (12.3 ± 2.1) than HCs (12.9 ± 1.7) with an odds ratio (OR) of 0.8 (95% confidence interval [CI] 0.70–0.98; *p* = 0.003). The strength of association was also confirmed at multivariate analysis (OR = 0.8, 95% CI 0.70–0.98; *p* = 0.003). Moreover, in PD patients, a statistically significant positive correlation was found between VRT-classification and MoCA scores (*r* = 0.330; *p* = 0.002).

**Conclusions:**

PD patients presented lower VR performance than HCs.

## 1. Introduction

Verbal reasoning (VR) is a cognitive ability considered essential for understanding, reasoning, and problem-solving using concepts conveyed by words, not limited to simply vocabulary comprehension or verbal fluency, leading to draw inferences from given information [1]. A “good” VR requires the involvement of many cognitive abilities including attention, working memory, abstraction, and categorization skills, crucial for cognitive performance and mainly related to executive functioning (EF) [2]. In particular, the ability to generalize principles from a specific and concrete one (abstraction), or to form concepts applying general rules (conceptualization and classification), represents cognitive skills interconnected with both EF and VR.

Actually, the impairment of these skills could lead, in practical application, to difficulties to learn from experiences in a logical, meaningful, and organized manner approaching daily life events as they were always unexpected as a “novel experience [3, 4].

Parkinson's disease (PD) is a neurodegenerative disorder characterized by both motor (rest tremor, bradykinesia, and rigidity) and non-motor symptoms. Among the latter, cognitive impairment, from mild cognitive decline to overt dementia, probably represents the most common and may conceivably occur at any disease stage, even before the occurrence of motor symptoms [5] or in combination with other non-motor symptoms [6]. Although all cognitive domains (i.e., episodic memory, attention, and language) may potentially be impaired, the frequent and predominant impairment of EF in PD has been constantly reported [7, 8]. However, even if EF and VR are interconnected, to the best of our knowledge, to date, only few studies have investigated VR abilities in PD patients [9].

The aim of the present study was to assess VR functioning in a group of non-demented PD patients and in a group of healthy controls (HCs).

## 2. Methods

Clinically established non-demented PD patients, diagnosed according to the Movement Disorders Society-PD criteria [10] and a group of HCs matched by age and global cognition, assessed with the administration of the Montreal Cognitive Assessment (MoCA), were enrolled in this study. As part of normal diagnostic work-up, to exclude secondary Parkinsonism and to support the diagnosis of PD, all PD patients performed a brain magnetic resonance imaging (MRI) [11]. Demographic, clinical, and pharmacological data were recorded. Levodopa equivalence daily dosage (LEDD) was calculated [12].

All participants referred to the Movement Disorders Center of the University Hospital “Policlinico-San Marco” of Catania, Italy. The study was approved by the ethical committee, and written informed consent was obtained from all enrolled subjects.

### 2.1. Clinical and Neuropsychological Assessment

All enrolled subjects underwent a standard neurological examination performed by movement disorders specialists. For PD patients, the severity of motor status was evaluated with the Unified Parkinson's Disease Rating Scale-Motor Examination section (UPDRS-ME) before levodopa intake. PD patients underwent the neuropsychological evaluation two hours after levodopa intake. The MoCA was administered to match PD patients and HCs according to general cognitive abilities. According to the Movement Disorders Society level I diagnostic criteria, patients obtaining a pathological MoCA score (MoCA < 26) were considered as suffering from mild cognitive impairment (MCI) [13].

VR was assessed with the verbal reasoning test (VRT), one of the few tests available to assess VR functioning. It is made up of 7 subsets, each of which has 7 items and investigates a different aspect of reasoning, so that the total items are 49. The subsets are absurdities, intruders, relationships, and differences; idiomatic expressions; family relations; and classification. Each subset has its own score, and the sum of all 7 subsets produces the total score, corrected by age and education [2].

### 2.2. Statistical Analysis

Data were analyzed using the STATA 16.0 software package (Stata Statistical Software: Release 16, StataCorp LLC, College Station, TX, USA). Quantitative variables were expressed as mean and standard deviation. Qualitative variables were expressed as number and percentage. The differences between means and the differences between proportions were evaluated by the *t*-test and the Chi-square test, respectively.

Logistic regression, univariate analysis, was performed to assess possible associations between PD and VRT scores (total and subset scores) analyzed as both categorical (pathological/normal values according to age and education adjusted cut-off) and continuous variables. Subsequently, multivariate analysis adjusted by sex, age, education, and disease duration, considered a priori confounders, was performed.

Finally, Pearson's correlation analysis was performed to investigate possible correlations between VRT and MoCA scores. Linear regression was then carried out, adjusting for sex, age, education, and disease duration, considered a priori confounders. The significance level was set at 0.05, and the 95% confidence intervals (CIs) were calculated.

## 3. Results

Eighty-seven PD patients (51 men; mean age 63.8 ± 7.9 years) and 87 HCs (46 men; mean age 63.7 ± 8.0 years) were enrolled. Demographic and general characteristics of the two groups were shown in Table 1.

According to MDS level I diagnostic criteria [13], out of the 87 subjects, 60 (68.9%) had MCI. A significantly higher percentage of PD patients presented pathological performance at the VRT subset classification (*n* = 53, 60.9%) than HCs (*n* = 36, 41.3%; odds ratio [OR] = 2.2; 95% CI 1.20–4.04; *p* = 0.010). At univariate analysis, PD patients presented a significantly lower score at the VRT subset classification (12.3 ± 2.1) than HCs (12.9 ± 1.7; OR = 0.8; 95% CI 0.70–0.98; *p* = 0.033; Table 2).

The strength of association was also confirmed at multivariate analysis adjusting for age, sex, education, and disease duration, considered a priori confounders (OR = 0.8, 95% CI 0.70–0.98; *p* = 0.033). No significant differences were found when comparing the two groups in the others VRT subset.

**Figure 1:**
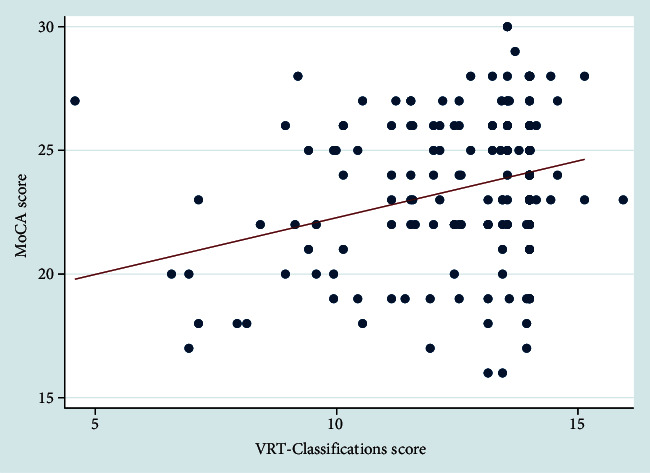
Pearson's correlation between MoCA scores and VRT-classification in patients with Parkinson's disease.

Moreover, only among PD patients, a statistically significant positive correlation was found between VRT-classification and MoCA scores (*r* = 0.330; *p* = 0.002; Figure 1). At the linear regression, this correlation was confirmed also adjusting for age, sex, education, and disease duration (coef = 0.289; 95% CI 0.02–0.55; *p* = 0.035).

## 4. Discussion

In the present study, PD patients, compared to age and global cognition matched HCs, presented a lower score in the VRT total score and in almost all subsets, even if the statistically significant difference was reached only for the “classification” one.

Moreover, in our study, only in PD patients, the score obtained in the VRT-classification subset was positively correlated with the MoCA score. Actually, the VRT-classification subset explores the same cognitive ability explored by the classification subset of the Frontal Assessment Battery, which has been recently correlated with the MoCA score in an Italian sample of healthy individuals [14].

*Classification* subset is related to the ability of the individual to group words into categories, to individualize the super-ordinate concept, detecting similarities between objects belonging to the same semantic category. This ability needs understanding the deeper relationship between objects, rather than simply collocating it hierarchical categories. In particular, in the VRT, the classification ability was assessed asking the participant to determine the category to which triplets of words belong (i.e., “What are Milan, Rome and Naples?”).

To the best of our knowledge, to date, only few studies have assessed VR in PD patients. In particular, Natsopoulos et al. compared 27 PD patients and 27 HCs in both deductive (i.e., syllogism) and inductive (i.e., classification) verbal reasoning reported impaired performance in PD patients, in particular in those patients with longer disease duration and more severe motor impairment [9].

Relatively, more recently, Young et al. [15] reported that PD patients (*n*.19) presented lower VR performance than HCs (*n* = 20), also supporting the interplay between VR and difficulties in everyday functioning based on real-life ecological situations (i.e., money management, food preparation, and medication use). Although our findings are in agreement with the studies performed by Natsopoulos et al. [9] and Young et al. [15], the data are difficult to compare due to methodological issues related to the different tests performed to assess VR and the small sample size of the previous studies.

From the neuroanatomical point of view, previous MRI studies associated the impairment in *classification* ability to the damage of the prefrontal cortex [16], whose thickness in PD patients has been frequently observed [17, 18].

Considering that VR is nothing but “understanding,” “reasoning,” and “thinking” using concepts conveyed by words (but which go beyond the “simple” understanding of the term), it could be deducted that its impairment could be associated with poor social relationships and quality of life. VR could in fact be considered crucial for daily life because it allows people to better understand the relationship between objects, concepts, and complicated events. As a matter of fact, deepening knowledge on this topic may be useful not only in terms of theory enhancing but also for a better clinical and rehabilitative approach empowering patient's interpersonal relationships.

We are aware that some limits should be taken into account when interpreting our data. Due to The hospital-based design of the study did not allow us to ruled out a selection bias related to the enrolment of more severe cases. However, the majority of PD patients enrolled in our study presented a short disease duration and a PD stage mild to moderate. Moreover, although patients with dementia were excluded from the study, not all the enrolled patients obtained “normal” age and education adjusted scores at the MoCA. However, it should be noted that PD and HCs were age and MoCA matched; furthermore, Santangelo et al. [19], considering that several items of the MoCA may be “culturally biased,” suggested to apply in the Italian population a reduced cut-off with respect to those proposed in the original version [20]. Finally, although the MoCA is considered particularly sensitive in detecting executive dysfunctions, the lack of a more “comprehensive” neuropsychological assessment did not allow us to clearly define the nature of the PD-specific VR deficit.

However, to the best of our knowledge, to date, our study is the larger study assessing VR in PD patients. Further studies are necessary to confirm our results and to clarify if VR impairment in PD patients could represent an early cognitive deficit.

## Figures and Tables

**Table 1:** Demographic and clinical characteristics.

	PD, *n* = 87	HCs, *n* = 87	*p*-Value
Sex (men)	51 (58.6)	46 (52.9)	0.445
Age (years)	63.8 ± 7.9	63.7 ± 8.0	0.939
Education (years)	11.8 ± 4.3	11.1 ± 3.9	0.206
MoCA score	23.4 ± 3.1	23.6 ± 2.9	0.410
Disease duration (years)	5.3 ± 3.2	—	—
UPDRS-ME score	27.1 ± 9.5	—	—
Hoeh and Yahr stage	2.2 ± 0.6	—	—
LEDD (mg/die)	423.9 ± 320.4	—	—

Data are expressed as mean ± standard deviation, and number and percentage. Abbreviations: PD = Parkinson's disease; HCs = healthy controls; UPDRS-ME = Unified Parkinson's Disease Rating Scale–Motor Examination; MoCA = Montreal Cognitive Assessment; LEDD = levodopa equivalent dosage.

**Table 2:** Verbal reasoning in PD and HCs. Univariate analysis.

	PD, *n* = 87	HCs, *n* = 87	OR	95% CI	*p*-Value
Absurdities	10.4 ± 2.4	10.8 ± 2.6	0.9	0.84–1.07	0.399
Absurdities, pathological performance <11.73	59 (67.8)	52 (59.8)	1.4	0.76–2.63	0.270
Intruders	10.5 ± 2.7	10.2 ± 3.0	1.0	0.93–1.14	0.546
Intruders pathological performance <12.63	58 (66.7)	65 (74.7)	0.7	0.35–1.30	0.245
Relationships	9.0 ± 3.4	9.4 ± 2.9	0.9	0.87–1.05	0.370
Relationships pathological performance <12.20	73 (83.9)	66 (75.8)	1.6	0.78–3.52	0.188
Differences	12.1 ± 1.9	13.1 ± 6.5	0.9	0.77–1.05	0.212
Differences pathological performance <12.91	48 (55.2)	42 (48.2)	1.3	0.72–2.39	0.363
Idiomatic expressions	11.3 ± 2.3	11.6 ± 2.2	0.9	0.81–1.06	0.314
Idiomatic expressions pathological performance <11.83	40 (45.9)	42 (48.3)	0.9	0.50–1.65	0.761
Family relationship	8.1 ± 4.0	8.7 ± 3.1	0.9	0.87–1.03	0.207
Family relationship pathological performance <11.0	68 (78.2)	77 (88.5)	0.5	0.20–1.06	0.071
Classifications	12.3 ± 2.1	12.9 ± 1.7	**0.8**	**0.70–0.98**	**0.033**
Classifications pathological performance <13.20	**53 (60.9)**	**36 (41.3)**	**2.2**	**1.20–4.04**	**0.010**
Total score	73.5 ± 12.5	76.4 ± 11.5	0.9	0.95–1.00	0.115
Total score pathological performance <84.27	67 (77.0)	64 (73.5)	1.2	0.60–2.40	0.589

Data are expressed as mean ± standard deviation, and number and percentage. Abbreviations: PD = Parkinson's disease; HC = healthy controls; OR = odds ratio; CI = confidence intervals.

p-value<0.005.

## Data Availability

Data supporting this research article are available from the corresponding author or first author on reasonable request.
